# Supplementary material for: Isolation and Characterization of a Novel Jumbo Phage from Leaf Litter Compost and Its Suppressive Effect on Rice Seedling Rot Diseases
Source: Viruses. 2021 Mar 31;13(4):591. doi: 10.3390/v13040591 (PMC8066314; doi:10.3390/v13040591)
Supplement: Supplementary file 1 [file viruses-13-00591-s001.zip › viruses-1156300-supplementary.pdf]

Sasaki et al., Fig. S1

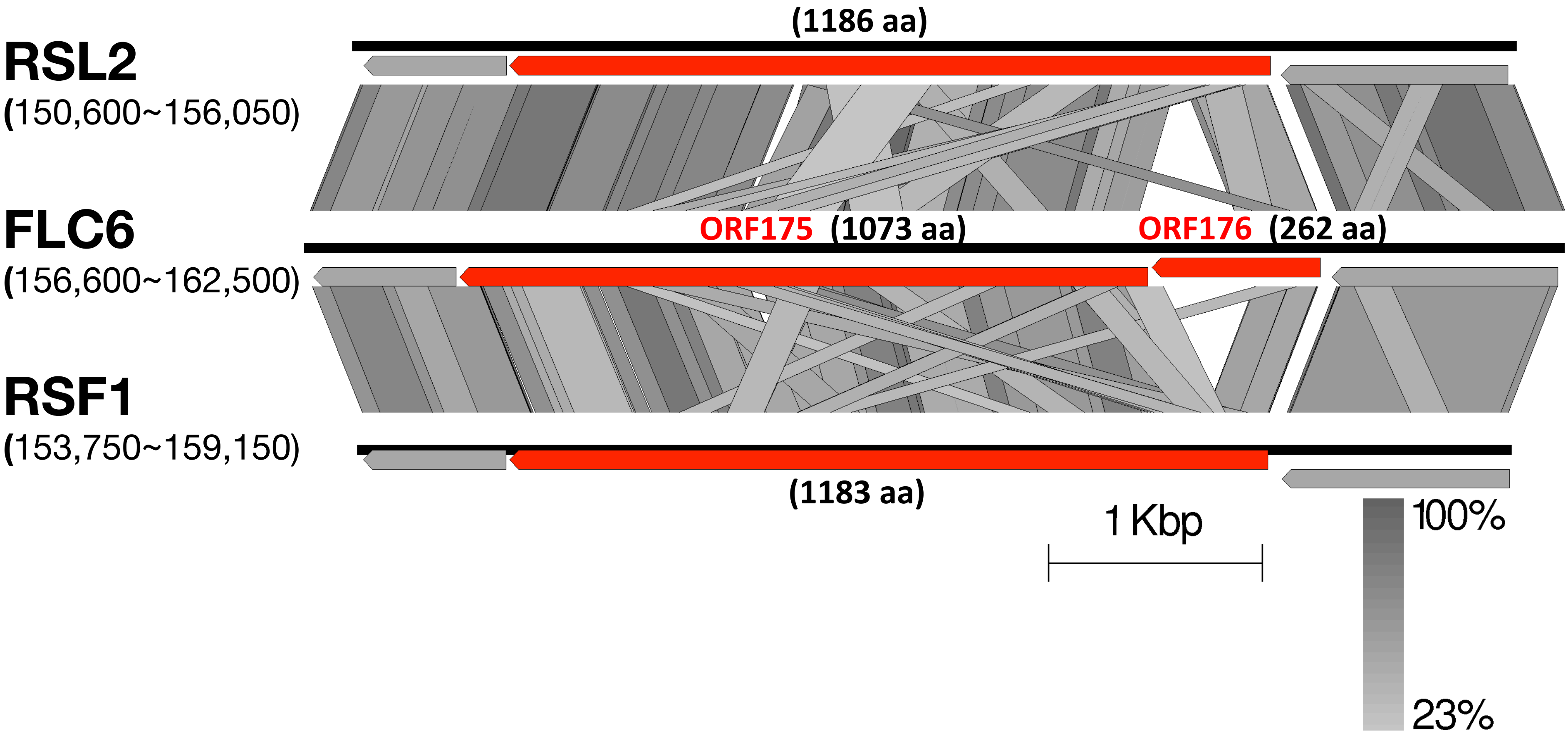

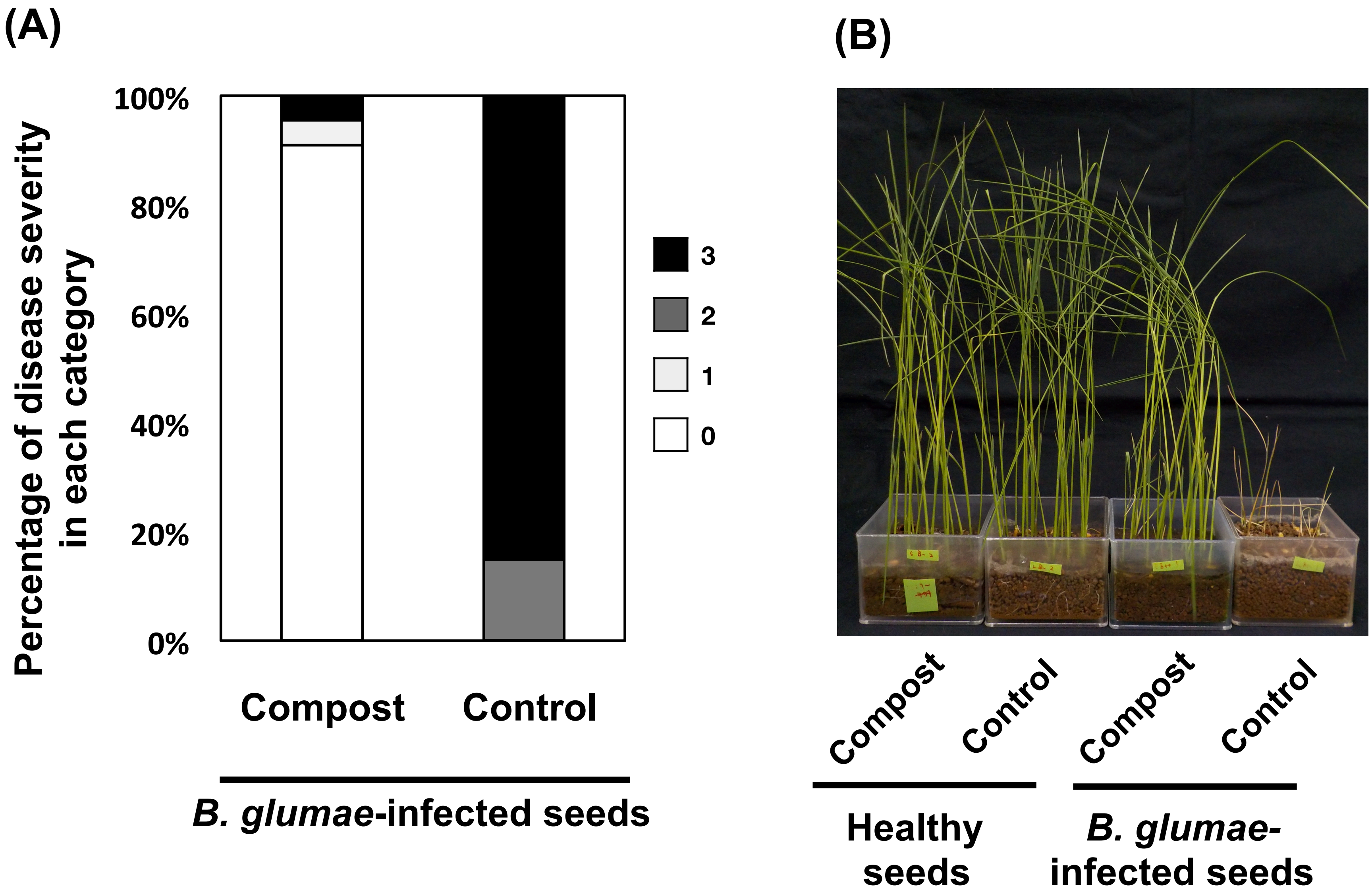
(A)
Percentage of disease severity in each category
100%
80%
60%
40%
20%
0%
3
2
1
0
Compost
Control
B. glumae-infected seeds
(B)
Compost
Control
Compost
Control
Healthy seeds
B. glumae-infected seeds

Table S1 Homology search of FLC6 gene products amino acid sequences.

FLC6	RSL2			RSF1			Closest hits other than RSL2 and RSF1[1)]		
	RefSeq accession	function	e value[2)]	RefSeq accession	function	e value[2)]	RefSeq accession	function	e value[2)]
**gp1**	YP_009212850.1	hypothetical protein	8.1E-110 *	YP_009207805.1	hypothetical protein	1.4E-87			
**gp2**	YP_009212851.1	hypothetical protein	5.1E-41 *	YP_009207806.2	hypothetical protein	4.4E-11			
**gp3**	YP_009212852.1	hypothetical protein	0 *	YP_009207807.1	hypothetical protein	0			
**gp4**	YP_009212853.1	hypothetical protein	7.1E-94 *	YP_009207808.1	hypothetical protein	1.3E-80			
**gp5**	YP_009212854.1	hypothetical protein	6.5E-56 *	YP_009207809.2	hypothetical protein	1.7E-52			
**gp6**	YP_009212855.1	hypothetical protein	3.8E-47	YP_009207810.1	hypothetical protein	8.5E-52 *			
**gp7**	YP_009212856.1	hypothetical protein	1.4E-41	YP_009207811.2	hypothetical protein	8E-79 *			
**gp8**	YP_009212857.1	hypothetical protein	5.8E-92 *	YP_009207812.2	hypothetical protein	2.4E-87			
**gp9**	YP_009212858.1	hypothetical protein	3.9E-55 *	YP_009207813.2	hypothetical protein	3.1E-38			
**gp10**	YP_009212859.1	hypothetical protein	3E-122	YP_009207814.2	hypothetical protein	1.9E-124 *			
**gp11**	YP_009212860.1	hypothetical protein	0	YP_009207815.1	hypothetical protein	0 *			
**gp12**	YP_009212861.1	hypothetical protein	0 *	YP_009207816.1	hypothetical protein	0			
**gp13**	YP_009212862.1	hypothetical protein	3.8E-98 *	YP_009207817.2	hypothetical protein	5.4E-41			
**gp14**	YP_009212863.1	hypothetical protein	5.6E-48 *	YP_009207818.1	hypothetical protein	6.2E-33			
**gp15**	no hits			YP_009241388.1	hypothetical protein	3.6E-11 *			
**gp16**	YP_009212864.1	hypothetical protein	2E-75 *	YP_009207819.1	hypothetical protein	3.4E-73			
**gp17**	YP_009212865.1	hypothetical protein	1.7E-35 *	YP_009207820.1	hypothetical protein	1.4E-14			
**gp18**	YP_009212866.1	hypothetical protein	0	YP_009207821.2	hypothetical protein	0 *			
**gp19**	YP_009212867.1	hypothetical protein	3E-123 *	YP_009207822.2	hypothetical protein	4.1E-102			
**gp20**	YP_009212868.1	hypothetical protein	2.4E-135 *	YP_009207824.1	hypothetical protein	1.2E-129			
**gp21**	YP_009212870.1	hypothetical protein	0 *	YP_009207826.1	hypothetical protein	0			
**gp22**	no hits			YP_009207827.1	hypothetical protein	1.8E-74 *			
**gp23**	no hits			YP_009207828.1	hypothetical protein	5.6E-17 *			
**gp24**	YP_009212871.1	hypothetical protein	6.9E-31 *	YP_009207829.1	hypothetical protein	7E-24			
**gp25**	YP_009212872.1	hypothetical protein	0 *	YP_009207830.1	hypothetical protein	0			
**gp26**	YP_009212873.1	hypothetical protein	0 *	YP_009207831.1	putative virion stractural protein	0			
**gp27**	YP_009212874.1	hypothetical protein	0 *	YP_009207832.2	putative virion structural protein	0			
**gp28**	YP_009212875.1	hypothetical protein	0 *	YP_009207833.1	hypothetical protein	0			
**gp29**	YP_009212876.1	hypothetical protein	0 *	YP_009207834.2	putative tail sheath	0			
**gp30**	YP_009212877.1	hypothetical protein	0 *	YP_009207835.1	putative major virion structural protein	0			
**gp31**	YP_009212878.1	hypothetical protein	0 *	YP_009207836.1	hypothetical protein	0			
**gp32**	YP_009212879.1	hypothetical protein	5E-77	YP_009207837.2	hypothetical protein	3.1E-80 *			
**gp33**	YP_009212880.1	hypothetical protein	1.3E-108 *	YP_009207838.1	hypothetical protein	1.1E-81			
**gp34**	YP_009212881.1	hypothetical protein	1.6E-51	YP_009207839.1	hypothetical protein	6E-72 *			
**gp35**	YP_009212882.1	hypothetical protein	0 *	YP_009207840.1	putative virion structural protein	7.9E-178			
**gp36**	YP_009212883.1	hypothetical protein	3E-177 *	YP_009207841.1	hypothetical protein	6.4E-169			
**gp37**	YP_009212884.1	hypothetical protein	0 *	YP_009207842.1	hypothetical protein	0			
**gp38**	YP_009212885.1	hypothetical protein	1.2E-59 *	YP_009207843.1	hypothetical protein	1.5E-46			
**gp39**	YP_009212886.1	hypothetical protein	0 *	YP_009207844.1	putative RNA polymerase beta subunit	0			
**gp40**	YP_009212887.1	hypothetical protein	0	YP_009207845.2	putative RNA polymerase beta prime subunit	0 *			
**gp41**	YP_009212888.1	hypothetical protein	0 *	YP_009207846.2	putative soluble lytic murein transglycosylase	0			
**gp42**	YP_009212889.1	hypothetical protein	0 *	YP_009207847.1	hypothetical protein	0			
**gp43**	YP_009212890.1	hypothetical protein	0 *	YP_009207848.1	hypothetical protein	0			
**gp44**	YP_009212891.1	hypothetical protein	5E-127 *	YP_009207849.2	hypothetical protein	2.4E-113			
**gp45**	YP_009212892.1	hypothetical protein	9.7E-85 *	YP_009207850.2	hypothetical protein	3.3E-66			
**gp46**	YP_009212893.1	hypothetical protein	1.8E-113 *	YP_009207851.2	hypothetical protein	7.9E-78			
**gp47**	YP_009212894.1	hypothetical protein	4.2E-60	YP_009207852.2	hypothetical protein	1.4E-88 *			
**gp48**	YP_009212895.1	hypothetical protein	0 *	YP_009207853.1	hypothetical protein	3.9E-134			
**gp49**	YP_009212896.1	hypothetical protein	3.3E-97 *	YP_009207854.1	hypothetical protein	1.1E-73			
**gp50**	YP_009212897.1	hypothetical protein	9.2E-166 *	YP_009207855.2	putative RNA polymerase beta subunit	5.4E-151			
**gp51**	YP_009212898.1	hypothetical protein	9E-106 *	YP_009207856.1	hypothetical protein	7.3E-93			
**gp52**	YP_009212899.1	hypothetical protein	0 *	YP_009207857.2	hypothetical protein	0			
**gp53**	YP_009212900.1	hypothetical protein	0 *	YP_009207858.1	hypothetical protein	0			
**gp54**	YP_009212901.1	hypothetical protein	5.3E-79 *	YP_009207859.2	putative transglycosylase SLT domain protein	1.3E-74			
**gp55**	no hits			YP_009207860.2	hypothetical protein	2.2E-48 *			
**gp56**	YP_009212903.1	hypothetical protein	0 *	YP_009207861.2	putative RNase H	0			
**gp57**	YP_009212904.1	hypothetical protein	6.2E-41 *	no hits					
**gp58**	YP_009212905.1	hypothetical protein	3.2E-86	YP_009207862.1	hypothetical protein	1.9E-95 *			
**gp59**	YP_009212906.1	hypothetical protein	0 *	YP_009207863.1	putative virion structural protein	0			
**gp60**	YP_009212907.1	hypothetical protein	4.5E-116 *	YP_009207864.1	hypothetical protein	6E-100			
**gp61**	no hits			no hits			WP_066022194.1	MULTISPECIES: hypothetical protein [Clostridium]	7.2.E-12 *
**gp62**	YP_009212909.1	hypothetical protein	2.5E-34 *	YP_009207865.2	hypothetical protein	4.4E-34			
**gp63**	YP_009212910.1	hypothetical protein	0 *	YP_009207867.1	putative SbcC-ATPase	0			
**gp64**	YP_009212911.1	hypothetical protein	0 *	YP_009207868.1	hypothetical protein	0			
**gp65**	no hits			no hits			no hits		
**gp66**	YP_009212912.1	hypothetical protein	0 *	YP_009207869.1	hypothetical protein	0			
**gp67**	YP_009212913.1	hypothetical protein	0 *	YP_009207870.1	hypothetical protein	0			
**gp68**	YP_009212914.1	hypothetical protein	0 *	YP_009207871.1	putative virion structural protein	0			
**gp69**	no hits			YP_009207872.1	putative GIY-YIG type nuclease	5.4E-142 *			
**gp70**	YP_009212915.1	hypothetical protein	5.4E-78 *	YP_009207873.1	putative cupin superfamily protein	4.6E-38			
**gp71**	YP_009212916.1	hypothetical protein	0	YP_009207874.1	putative Fe-S oxidoreductase	0 *			
**gp72**	YP_009212917.1	hypothetical protein	8.9E-132 *	YP_009207875.1	hypothetical protein	1.2E-98			
**gp73**	YP_009212918.1	hypothetical protein	0 *	YP_009207876.1	hypothetical protein	0			
**gp74**	YP_009212919.1	hypothetical protein	0 *	YP_009207877.2	hypothetical protein	1.4E-159			
**gp75**	YP_009212920.1	hypothetical protein	0 *	YP_009207878.2	hypothetical protein	1.2E-170			
**gp76**	YP_009212921.1	hypothetical protein	0 *	YP_009207879.1	putative radical SAM superfamily	0			
**gp77**	YP_009212922.1	hypothetical protein	3.3E-74 *	YP_009207881.1	putative dihydrofolate reductase	2.9E-71			
**gp78**	YP_009212923.1	hypothetical protein	3.1E-29 *	YP_009207882.1	putative dihydrofolate reductase	3.4E-25			
**gp79**	YP_009212924.1	hypothetical protein	0 *	YP_009207883.2	putative Fe-S oxidoreductase	0			
**gp80**	YP_009212925.1	hypothetical protein	4.6E-129 *	YP_009207884.1	putative 2OG-Fe(II) oxygenase	3.4E-118			
**gp81**	YP_009212926.1	hypothetical protein	0 *	YP_009207885.2	putative radical SAM superfamily	4.1E-176			
**gp82**	YP_009212927.1	hypothetical protein	1.5E-116 *	YP_009207886.2	putative 2OG-Fe(II) oxygenase	6.7E-77			
**gp83**	YP_009212928.1	hypothetical protein	6.1E-138 *	YP_009207887.1	hypothetical protein	3.4E-114			
**gp84**	YP_009212929.1	hypothetical protein	2.1E-140 *	YP_009207888.1	hypothetical protein	1.4E-119			
**gp85**	YP_009212930.1	hypothetical protein	2.7E-61	YP_009207889.2	hypothetical protein	7.9E-75 *			
**gp86**	no hits			YP_009207891.1	hypothetical protein	2E-36 *			
**gp87**	YP_009212931.1	hypothetical protein	1.4E-141 *	YP_009207892.1	hypothetical protein	7.1E-69			
**gp88**	YP_009212908.1	hypothetical protein	6.8E-11	YP_009207893.2	putative concanavalin A-like protein	2.7E-85 *			
**gp89**	no hits			no hits			WP_091507268.1	glycosyl hydrolase [Amycolatopsis sacchari]	8.1.E-08 *
**gp90**	YP_009212933.1	hypothetical protein	4E-166 *	YP_009207895.1	hypothetical protein	3.2E-162			
**gp91**	no hits			YP_009207896.2	hypothetical protein	2.5E-51 *			
**gp92**	YP_009212934.1	hypothetical protein	0 *	YP_009207898.2	hypothetical protein	2E-154			
**gp93**	YP_009212935.1	hypothetical protein	6.4E-48	YP_009207899.1	hypothetical protein	1E-48 *			
**gp94**	YP_009212936.1	hypothetical protein	3.8E-70 *	YP_009207900.1	hypothetical protein	4.3E-65			
**gp95**	YP_009212937.1	hypothetical protein	4.1E-114 *	YP_009207901.1	hypothetical protein	2E-84			
**gp96**	YP_009212938.1	hypothetical protein	1.3E-140 *	YP_009207902.2	hypothetical protein	8.9E-95			
**gp97**	YP_009212939.1	hypothetical protein	0 *	YP_009207903.1	hypothetical protein	0			
**gp98**	YP_009212940.1	hypothetical protein	0 *	YP_009207904.2	putative virion structural protein	0			
**gp99**	YP_009212941.1	hypothetical protein	0 *	YP_009207905.1	putative virion structural protein	0			
**gp100**	no hits			no hits			no hits		
**gp101**	no hits			no hits			WP_066019941.1	MULTISPECIES: LamG domain-containing protein [Clostridium]	2.2.E-08 *
**gp102**	no hits			no hits			no hits		
**gp103**	YP_009212943.1	hypothetical protein	1E-40	YP_009207908.2	hypothetical protein	7.5E-70 *			
**gp104**	YP_009212943.1	hypothetical protein	1.6E-42 *	YP_009207908.2	hypothetical protein	3.1E-25			
**gp105**	YP_009212944.1	hypothetical protein	0 *	YP_009207909.1	putative DNA ligase	0			
**gp106**	no hits			YP_009207910.1	hypothetical protein	1.7E-40 *			
**gp107**	YP_009212946.1	hypothetical protein	4.6E-108 *	YP_009207911.1	hypothetical protein	1.4E-50			
**gp108**	YP_009212947.1	hypothetical protein	1.5E-60 *	YP_009207912.2	hypothetical protein	7.5E-48			
**gp109**	YP_009212948.1	hypothetical protein	4.8E-129 *	YP_009207913.2	hypothetical protein	1E-120			
**gp110**	YP_009212950.1	hypothetical protein	5.9E-57 *	YP_009207914.2	hypothetical protein	7.3E-49			
**gp111**	YP_009212951.1	hypothetical protein	1.6E-96 *	YP_009207915.1	hypothetical protein	4.9E-80			
**gp112**	YP_009212952.1	hypothetical protein	6.3E-41 *	YP_009207916.2	hypothetical protein	2.8E-21			
**gp113**	YP_009212953.1	hypothetical protein	0 *	YP_009207917.1	hypothetical protein	0			
**gp114**	YP_009212954.1	hypothetical protein	0 *	YP_009207918.1	hypothetical protein	0			
**gp115**	YP_009212955.1	hypothetical protein	1.9E-142 *	YP_009207919.1	hypothetical protein	1.5E-86			
**gp116**	YP_009212956.1	hypothetical protein	6.3E-55 *	YP_009207920.2	hypothetical protein	6.1E-37			
**gp117**	YP_009212958.1	hypothetical protein	2.7E-172 *	no hits					
**gp118**	YP_009212959.1	hypothetical protein	0 *	YP_009207921.1	putative ribonucleotide reductase beta subunit	0			
**gp119**	YP_009212960.1	hypothetical protein	0 *	YP_009207922.1	putative ribonucleotide reductase alpha subunit	0			
**gp120**	YP_009212961.1	hypothetical protein	1.4E-52 *	YP_009207923.2	putative anaerobic ribonucleotide diphosphate reductase subunit H	1.1E-36			
**gp121**	YP_009212962.1	hypothetical protein	4.9E-98 *	YP_009207924.1	hypothetical protein	3.5E-91			
**gp122**	YP_009212963.1	hypothetical protein	0 *	YP_009207925.2	hypothetical protein	0			
**gp123**	YP_009212964.1	hypothetical protein	0 *	YP_009207926.2	putative RNA polymerase beta subunit	0			
**gp124**	YP_009212965.1	hypothetical protein	0 *	YP_009207927.1	hypothetical protein	0			
**gp125**	YP_009212966.1	hypothetical protein	0 *	YP_009207928.1	putative major head protein	0			
**gp126**	YP_009212967.1	hypothetical protein	1.1E-152 *	YP_009207929.1	hypothetical protein	4.6E-135			

**gp127**	YP_009212968.1	hypothetical protein	0 *	YP_009207930.2	putative DnaB helicase	0			
**gp128**	no hits			no hits			WP_097190676.1	GIY-YIG nuclease family protein [Burkholderia sp. OK806]	3.5.E-08 *
**gp129**	no hits			no hits			WP_087940946.1	ATP-binding protein [Algoriphagus faecimaris]	1.3.E-30 *
**gp130**	no hits			no hits			no hits		
**gp131**	YP_009212970.1	hypothetical protein	1.6E-43 *	YP_009207932.1	hypothetical protein	5.6E-23			
**gp132**	no hits			YP_009207933.2	hypothetical protein	1.2E-17 *			
**gp133**	YP_009212971.1	hypothetical protein	1.3E-66 *	YP_009207934.1	hypothetical protein	8.6E-21			
**gp134**	YP_009212972.1	hypothetical protein	7.6E-44	YP_009207935.1	hypothetical protein	1.8E-64 *			
**gp135**	YP_009212973.1	hypothetical protein	1.1E-62 *	YP_009207937.2	hypothetical protein	7.8E-48			
**gp136**	YP_009212974.1	hypothetical protein	2.4E-29 *	YP_009207938.2	hypothetical protein	1.1E-11			
**gp137**	YP_009212975.1	hypothetical protein	7.3E-30 *	YP_009207939.1	hypothetical protein	1.2E-16			
**gp138**	YP_009212976.1	hypothetical protein	3.9E-129 *	YP_009207943.1	hypothetical protein	3.2E-115			
**gp139**	YP_009212978.1	hypothetical protein	0 *	YP_009207944.2	hypothetical protein	8.5E-140			
**gp140**	YP_009212979.1	hypothetical protein	1.5E-57 *	YP_009207945.1	hypothetical protein	8.4E-22			
**gp141**	YP_009212980.1	hypothetical protein	7.3E-33 *	YP_009207946.2	hypothetical protein	2E-27			
**gp142**	YP_009212981.1	hypothetical protein	0 *	YP_009207947.2	hypothetical protein	1.9E-141			
**gp143**	no hits			YP_009207948.2	hypothetical protein	8.6E-67 *			
**gp144**	YP_009212982.1	hypothetical protein	0 *	YP_009207949.1	hypothetical protein	0			
**gp145**	YP_009212983.1	hypothetical protein	2.5E-92 *	YP_009207950.1	hypothetical protein	7.9E-69			
**gp146**	YP_009212984.1	hypothetical protein	3E-85 *	YP_009207951.1	hypothetical protein	1.5E-74			
**gp147**	YP_009212985.1	hypothetical protein	1.6E-24 *	YP_009207952.1	hypothetical protein	5.9E-24			
**gp148**	no hits			no hits			no hits		
**gp149**	YP_009212986.1	hypothetical protein	1.3E-73 *	YP_009207953.1	hypothetical protein	5.3E-62			
**gp150**	YP_009212987.1	hypothetical protein	6.2E-100 *	YP_009207954.1	hypothetical protein	5.5E-47			
**gp151**	YP_009212988.1	hypothetical protein	3.4E-23 *	YP_009207955.1	hypothetical protein	3.6E-17			
**gp152**	YP_009212989.1	hypothetical protein	7.8E-178 *	YP_009207956.1	hypothetical protein	9.8E-82			
**gp153**	no hits			no hits			no hits		
**gp154**	YP_009212990.1	hypothetical protein	1.3E-146 *	YP_009207957.1	hypothetical protein	5.6E-114			
**gp155**	YP_009212991.1	hypothetical protein	7.1E-48 *	no hits					
**gp156**	YP_009212992.1	hypothetical protein	2.5E-97 *	YP_009207959.2	hypothetical protein	6E-58			
**gp157**	YP_009212993.1	hypothetical protein	4.7E-36 *	YP_009207960.1	hypothetical protein	9.6E-10			
**gp158**	no hits			no hits			no hits		
**gp159**	YP_009212994.1	hypothetical protein	7.5E-66 *	no hits					
**gp160**	YP_009212995.1	hypothetical protein	3E-124 *	YP_009207961.1	hypothetical protein	1.1E-103			
**gp161**	YP_009212996.1	hypothetical protein	1.1E-72 *	YP_009207962.1	hypothetical protein	5.2E-69			
**gp162**	YP_009212998.1	hypothetical protein	4.9E-111 *	YP_009207964.2	putative haloacid reductase-like hydrolase	1.6E-105			
**gp163**	no hits			no hits			no hits		
**gp164**	YP_009212999.1	hypothetical protein	1.2E-105 *	YP_009207965.1	hypothetical protein	1.5E-91			
**gp165**	YP_009213000.1	hypothetical protein	2.9E-131 *	YP_009207966.2	hypothetical protein	4.6E-118			
**gp166**	YP_009213001.1	hypothetical protein	4.4E-86 *	YP_009207967.2	hypothetical protein	4.7E-59			
**gp167**	no hits			no hits			no hits		
**gp168**	YP_009213002.1	hypothetical protein	0 *	YP_009207968.1	putative thymidylate synthase	0			
**gp169**	YP_009213003.1	hypothetical protein	3E-46 *	YP_009207969.1	hypothetical protein	2.3E-39			
**gp170**	no hits			no hits			no hits		
**gp171**	no hits			no hits			no hits		
**gp172**	YP_009213004.1	hypothetical protein	0 *	YP_009207970.1	hypothetical protein	0			
**gp173**	YP_009213005.1	hypothetical protein	3.4E-92 *	YP_009207971.2	hypothetical protein	4.4E-69			
**gp174**	YP_009213006.1	hypothetical protein	2.2E-129	YP_009207972.1	hypothetical protein	1.8E-135 *			
**gp175**	YP_009213007.1	hypothetical protein	0 *	YP_009207973.2	putative tail fiber protein	0			
**gp176**	YP_009213007.1	hypothetical protein	1.2E-43	YP_009207973.2	putative tail fiber protein	6.5E-44 *			
**gp177**	YP_009213008.1	hypothetical protein	0 *	YP_009207974.1	hypothetical protein	0			
**gp178**	YP_009213009.1	hypothetical protein	1.7E-53 *	YP_009207975.1	hypothetical protein	3.3E-45			
**gp179**	no hits			no hits			YP_008433367.1	hypothetical protein PaBG_00036 [Pseudomonas phage PaBG]	1.5.E-19 *
**gp180**	YP_009213011.1	hypothetical protein	1.9E-86 *	YP_009207977.1	hypothetical protein	1.5E-44			
**gp181**	YP_009213012.1	hypothetical protein	0 *	YP_009207978.1	hypothetical protein	0			
**gp182**	YP_009213013.1	hypothetical protein	7.6E-122 *	YP_009207979.2	hypothetical protein	2.1E-100			
**gp183**	YP_009213014.1	hypothetical protein	0 *	YP_009207980.1	putative virion stractural protein	0			
**gp184**	YP_009213015.1	hypothetical protein	0 *	YP_009207981.2	hypothetical protein	0			
**gp185**	YP_009213016.1	hypothetical protein	1.1E-91 *	no hits					
**gp186**	YP_009213017.1	hypothetical protein	3.8E-57 *	YP_009207982.2	hypothetical protein	3.9E-09			
**gp187**	YP_009213018.1	hypothetical protein	0 *	YP_009207983.1	hypothetical protein	0			
**gp188**	YP_009213019.1	hypothetical protein	0	YP_009207984.1	hypothetical protein	0 *			
**gp189**	YP_009213020.1	hypothetical protein	1.7E-140 *	YP_009207985.1	hypothetical protein	1.6E-124			
**gp190**	YP_009213021.1	hypothetical protein	0 *	YP_009207986.1	putative virion stractural protein	0			
**gp191**	YP_009213022.1	hypothetical protein	0 *	YP_009207987.1	hypothetical protein	0			
**gp192**	YP_009213023.1	hypothetical protein	0 *	YP_009207988.2	putative virion stractural protein	8E-139			
**gp193**	YP_009213024.1	hypothetical protein	0 *	YP_009207989.1	putative virion stractural protein	0			
**gp194**	YP_009213025.1	hypothetical protein	0 *	YP_009207990.1	hypothetical protein	0			
**gp195**	YP_009213026.1	hypothetical protein	3.5E-108 *	YP_009207991.1	hypothetical protein	4.4E-93			
**gp196**	YP_009213027.1	hypothetical protein	0 *	YP_009207992.2	hypothetical protein	0			
**gp197**	YP_009213028.1	hypothetical protein	0 *	YP_009207993.2	hypothetical protein	0			
**gp198**	YP_009213029.1	hypothetical protein	5.2E-41 *	YP_009207994.1	putative thymidylate kinase	1.5E-37			
**gp199**	YP_009212850.1	hypothetical protein	6.1E-31	YP_009207995.2	hypothetical protein	1.1E-69 *			
**gp200**	YP_009213031.1	hypothetical protein	4.6E-162 *	YP_009207996.2	hypothetical protein	1.1E-143			
**gp201**	YP_009213035.1	hypothetical protein	1.9E-130 *	YP_009207997.1	hypothetical protein	3.9E-125			
**gp202**	YP_009213036.1	hypothetical protein	7.2E-154 *	YP_009207998.1	hypothetical protein	1.7E-138			
**gp203**	YP_009213037.1	hypothetical protein	2.3E-83 *	YP_009207999.2	hypothetical protein	1.9E-66			
**gp204**	YP_009213038.1	hypothetical protein	0 *	YP_009208000.1	hypothetical protein	0			
**gp205**	YP_009213039.1	hypothetical protein	2.2E-149 *	YP_009208001.1	hypothetical protein	2.4E-92			
**gp206**	YP_009213040.1	hypothetical protein	0 *	YP_009208002.2	hypothetical protein	0			
**gp207**	YP_009213041.1	hypothetical protein	0 *	YP_009208003.1	putative RNA polymerase beta prime subunit	0			
**gp208**	YP_009213042.1	hypothetical protein	3.7E-112 *	YP_009208004.2	hypothetical protein	2.1E-101			
**gp209**	YP_009213043.1	hypothetical protein	0 *	YP_009208005.1	hypothetical protein	3.4E-97			
**gp210**	YP_009213044.1	hypothetical protein	1.1E-88 *	YP_009208006.2	hypothetical protein	1.6E-47			
**gp211**	YP_009213045.1	hypothetical protein	1.3E-59 *	YP_009208007.2	hypothetical protein	1.6E-28			
**gp212**	no hits			no hits			no hits		
**gp213**	YP_009213048.1	hypothetical protein	1.2E-65 *	YP_009208009.1	hypothetical protein	1.4E-40			
**gp214**	no hits			YP_009208010.1	hypothetical protein	2.1E-56 *			
**gp215**	YP_009213050.1	hypothetical protein	1.2E-116 *	YP_009208012.1	hypothetical protein	1.5E-90			
**gp216**	YP_009213051.1	hypothetical protein	8.1E-144 *	YP_009208013.2	hypothetical protein	7.2E-127			
**gp217**	YP_009213052.1	hypothetical protein	5.8E-57 *	YP_009208014.1	hypothetical protein	2.3E-22			
**gp218**	YP_009213053.1	hypothetical protein	1.6E-58 *	no hits					
**gp219**	no hits			no hits			no hits		
**gp220**	YP_009213054.1	hypothetical protein	3.5E-20 *	YP_009208015.1	hypothetical protein	4.4E-16			
**gp221**	YP_009213055.1	hypothetical protein	0 *	YP_009208016.2	hypothetical protein	0			
**gp222**	YP_009213056.1	hypothetical protein	4.3E-128 *	YP_009208017.1	hypothetical protein	8.4E-91			
**gp223**	YP_009213057.1	hypothetical protein	0 *	YP_009208018.2	putative RNA polymerase beta prime subunit	0			
**gp224**	YP_009213058.1	hypothetical protein	0 *	YP_009208019.2	putative RNA polymerase beta subunit	0			
**gp225**	YP_009213059.1	hypothetical protein	8.5E-73 *	YP_009208020.1	hypothetical protein	1.3E-63			
**gp226**	YP_009213060.1	hypothetical protein	0 *	YP_009208021.1	hypothetical protein	0			
**gp227**	YP_009213061.1	hypothetical protein	0 *	YP_009208022.1	hypothetical protein	0			
**gp228**	YP_009213062.1	hypothetical protein	0 *	YP_009208023.2	hypothetical protein	0			
**gp229**	YP_009213063.1	hypothetical protein	8.5E-113 *	YP_009208024.1	hypothetical protein	6.9E-107			
**gp230**	YP_009213064.1	hypothetical protein	0 *	YP_009208025.2	hypothetical protein	0			
**gp231**	no hits			no hits			no hits		
**gp232**	YP_009213065.1	hypothetical protein	2.5E-40	YP_009208026.1	hypothetical protein	8.1E-42 *			
**gp233**	YP_009213066.1	hypothetical protein	0 *	YP_009208027.2	hypothetical protein	0			
**gp234**	YP_009213067.1	hypothetical protein	1.3E-159 *	YP_009208028.1	hypothetical protein	5.2E-138			
**gp235**	YP_009213068.1	hypothetical protein	4.5E-93 *	YP_009208029.2	hypothetical protein	2.5E-59			
**gp236**	YP_009213069.1	hypothetical protein	1.1E-154 *	YP_009208030.1	hypothetical protein	2.5E-125			
**gp237**	YP_009213070.1	hypothetical protein	0 *	YP_009208031.2	putative RNA polymerase beta prime subunit	0			
**gp238**	YP_009213071.1	hypothetical protein	1E-46 *	YP_009208032.2	hypothetical protein	6.3E-09			
**gp239**	YP_009213072.1	hypothetical protein	0 *	YP_009208033.2	hypothetical protein	0			
**gp240**	YP_009213073.1	hypothetical protein	0 *	YP_009208034.1	hypothetical protein	0			
**gp241**	no hits			no hits			no hits		

1) It is shown If the closest hit is neither the gene products of RSL2 nor RSF1.
2) "*" means the lowest e value.
